# The relationship between traits optimism and anxiety and health-related quality of life in patients hospitalized for chronic diseases: data from the SATISQOL study

**DOI:** 10.1186/1477-7525-11-134

**Published:** 2013-08-05

**Authors:** Sabrina Kepka, Cédric Baumann, Amélie Anota, Gaelle Buron, Elisabeth Spitz, Pascal Auquier, Francis Guillemin, Mariette Mercier

**Affiliations:** 1 University of Franche-Comté, UPRES EA 3181, Besançon, France; 2 Emergency and Intensive Care Department CHU, University Hospital Jean Minjoz, 1 Bvd Fleming, Besançon 25030, France; 3 Université de Lorraine, Université Paris Descartes, EA 4360 APEMAC, Nancy, France; 4 INSERM, CIC-EC CIE6, Nancy, France; 5 CHU de Nancy, Service épidémiologie et évaluation clinique, Nancy, France; 6 University Hospital Jean Minjoz, Quality of Life and Cancer Plateform, Besançon, France; 7 Université de Lorraine, Université Paris Descartes, EA 4360 APEMAC-EPSAM, Metz, France; 8 University of Mediterranee, UPRES EA 3279, Marseille, France

**Keywords:** Quality of life, Chronic diseases, Validated questionnaires, Anxiety, Optimism

## Abstract

**Background:**

The impact of psychological factors is often taken into account in the evaluation of quality of life. However, the effect of optimism and trait anxiety remains controversial and they are rarely studied simultaneously. We aimed to study the effect of this factor on health-related quality of life (HRQOL) of patients after a hospitalization in relation with their chronic disease.

**Methods:**

Using cross-sectional data from the SATISQOL cohort, we conducted a multicentric study, including patients hospitalized for an intervention in connection with their chronic disease. Six months after hospitalization, patients completed a generic HRQOL questionnaire (SF-36), and the STAI and LOT-R questionnaires to evaluate optimism and trait anxiety. We studied the effect of each trait on HRQOL separately, and simultaneously, taking account of their interaction in 3 models, using an ANOVA.

**Results:**

In this study, 1529 patients were included in three participating hospitals and there existed wide diversity in the chronic diseases in our population. The HRQOL score increased for all dimensions of SF36 between 15,8 and 44,5 when the level of anxiety decreased (p < 0.0001) for the model 1, assessing the effect of anxiety on HRQOL and increased for all dimensions of SF36 between 3.1 and 12.7 with increasing level of optimism (< 0.0001) in the model 2 assessing the effect of optimism on HRQOL. In the model 3, assessing the effect of both anxiety and optimism on HRQOL, and their interaction, the HRQOL score for all dimensions of the SF36 increased when the level of anxiety decreased (p < 0.0001). It increased with increasing level of optimism (p < 0.006) in the model for all dimensions of SF36 except the Role Physical dimension. In this model, interaction between anxiety and optimism was significant for the Social Functioning dimension (p = 0.0021).

**Conclusions:**

Optimism and trait anxiety appeared to be significantly correlated with HRQOL. Furthermore, an interaction existed between the trait anxiety and optimism for some dimensions of SF36. Contrary to optimism, it seems essential to evaluate trait anxiety in future studies about HRQOL, since it could represent a confounding factor.

## Background

For the follow-up of chronic diseases, it is necessary to develop indicators that can easily be assessed, such as the measure of the health-related quality of life (HRQOL), which is governed by specific guidelines for implementation [1,2]. HRQOL as an indicator provides essential information to the clinicians to estimate the efficiency of their therapeutic and preventive actions [3,4]. Patients affected by chronic disease have a particular profile, due to their recourse to regular care and the necessity to adapt to their disease, and this can have consequences on HRQOL assessment. Thus, chronic diseases can generate psychological distress [5] and can be associated with a lower HRQOL [6]. The conceptual framework for our study is a variation on Broffenbrenner’s ecological model [7], proposed by McLeroy [8], and explains the multiple levels of influence on health outcomes at both individual and environmental characteristics in HRQoL. The McLeroy model indicates five levels of influence: (a) intrapersonal factors (characteristics of individual such as personality traits, knowledge, attitudes, behavior, self-concept, skills, etc.), (b) interpersonal factors (formal and informal social support systems, including the family, work group, and friendship networks), (c) institutional factors (social institutions, organizations such as schools and healthcare facilities), (d) community factors (relationships among institutions and informal social networks in a defined area), and (e) public policy (local, state, and national laws and policies). For our proposed model, we considered only the influence at the individual level.

Some determinants of HRQOL such as gender, type of disease, age or socio-demographic characteristics (e.g. level of education, professional activity…) have been clearly identified in the literature. According to the original conceptual model of Wilson [9] reviewed by Ferrans [10], characteristics pertaining to both the individual and the environment can have an impact on the five major domains of HRQOL, namely biological and physiological factors, symptoms status, functional status, general health perceptions, and overall HRQOL. The effect of psychological characteristics has also been often evoked, but still warrants further exploration [11]. In this study we focus on the personal characteristics, particularly anxiety and optimism. Optimism and trait anxiety are characteristics inherent to every individual, and do not change over time or according to events with which the individual is confronted. Scheier and Carver theorized that the “disposition” towards optimism could be called “dispositional optimism” and proposed the notion of a measure for optimism [12,13]. They defined it as a relatively stable feature of the personality, which has important consequences on the way a person regulates their actions in the face of difficulties or stressful situations. For anxiety, Spielberger distinguished the notions of « state » and « trait » anxiety. He characterized trait anxiety as relatively stable individual differences in the tendency towards anxiety [14]. This tendency would be consistent according to different types of stressful situations and would be stable over time [14,15].

Psychological factors could be considered as items determining the quality of life [6,16]. Furthermore, HRQOL and anxiety or optimism can influence how patients accept a diagnosis, and can be used as an outcome to evaluate the efficiency of a particular therapeutic approach [17,18]. However, the relation between these factors has never been specifically addressed, and available data in the literature do not yield a consensus regarding the role of these psychological factors in HRQOL.

Many studies have underlined the importance of optimism in the evaluation of HRQOL [4,19-23] but its impact remains controversial. Optimistic patients may have coping strategies characterized by better acceptance of the disease, and this can contribute to a lower risk of certain chronic diseases and as a result, better HRQOL [24-27]. Using negative coping has been reported to be associated with low levels of optimism and a high level of anxiety [28]. Anxiety is thus associated with a lower HRQOL [29-34]. Accordingly, pessimistic patients could have a lower HRQOL, exacerbated by anxiety or depression [21,35]. On the other hand, an association between optimism and anxiety or depression and HRQOL may no longer be significant after adjusting for anxiety, depression and socio-demographics variables [23]. Thus, the role of each of these factors has often been taken into account separately in the evaluation of HRQOL, and few studies have evaluated both simultaneously [23]. Furthermore, the results of studies published to date are divergent regarding the role of each factor. If these two factors are related to HRQOL, they are thus potential confounding factors, and it is therefore necessary to know the effect of each trait and to take it into account in the evaluation of HRQOL.

On the basis of Wilson’s [9] and Ferrans models [10], the objective of this study is to clarify the relationships between trait anxiety, optimism and HRQOL. We hypothesized that the model has three causal pathways that contribute to the outcome variable, HRQOL: the anxiety-trait as a predictor (α), the impact of optimism as a moderator (β), and the interaction of these two (α and β) [36]. The moderator hypothesis is supported if the interaction is significant. There may also be significant main effects for the predictor and the moderator, but these are not directly relevant conceptually to testing the moderator hypothesis. We aimed to evaluate the relation between HRQOL and the traits optimism and anxiety among patients after hospitalization in relation to their chronic disease.

## Methods

### Design

**Figure 1:**
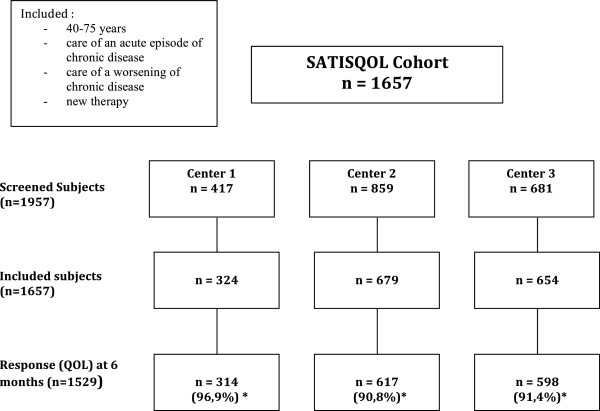
Flowchart of the SATISQOL cohort population.

This study was performed using cross-sectional data from patients with chronic diseases included in the SATISQOL (SATisfaction and Quality Of Life) cohort study (Figure 1).

### Population and sample

Patients were recruited during a stay in any of the 3 participating hospitals, in either medical or surgical departments. Patients were eligible if they were aged from 40 to 75 years old, hospitalized for the care of a chronic disease (defined as a disease ongoing for more than 6 months, and confirmed by a validation committee), and if the motive for hospitalization was an acute episode of the chronic disease, management of acute worsening of the disease, or initiation of new therapy for the chronic disease. We thus included any hospitalization for a medical or surgical intervention in connection with the chronic disease. Patients were excluded if they were hospitalized for a diagnostic assessment.

For the SATISQOL study, a HRQOL questionnaire was sent to each patient at 6 months and one year after discharge. Optimism and anxiety questionnaires were sent at 6 months only because they were considered to be stable over time. Clinical research assistants collected socio-demographic data and patient characteristics in each center. Subjects were informed about the study and written informed consent was obtained from all participants.

The SATISQOL study was approved by the national Institutional Review Board and the national committee for data protection (CCTIRS 07.212 and CNIL 1248560).

The SATISQOL cohort project was supported by an IRESP (Institut de recherche en santé publique) grant from Inserm, and a national hospital research grant from French Ministry of Health (PHRC, Programme Hospitalier de Recherche Clinique).

### Data collection

The primary endpoint of our study was HRQOL at 6 months.

### Health-related quality of life (HRQOL)

HRQOL was assessed using the validated French version of the MOS Short-Form 36 [37-39]. It includes 36 items, distributed in 8 dimensions: Physical Functioning (PF), Role Physical (RP), Bodily Pain (BP), Vitality, (VT) Social Functioning (SF), Role Emotional (RE), Mental Health (MH) and General Health (GH). A score was calculated for each dimension if more than half the items were completed. These scores were transformed to obtain a value between 0 (worst possible HRQOL) and 100 (best possible HRQOL) for each dimension.

### Anxiety

Trait anxiety was estimated by the STAI (State Trait Anxiety Inventory) validated in French. Each answer to the 20 items of the questionnaire scored 1 to 4. A global score ranging from 20 (the lowest level of anxiety) to 80 (the highest level of anxiety) was calculated if there were less than 20% missing data, corresponding to at least 17 items completed.

### Optimism

Optimism was estimated by the LOT-R (Life Orientation Test Revised) questionnaire. It included 6 statements of personal evaluation concerning general expectations relative to positive or negative consequences. Four statements were included as decoys. Every item scored 0 to 4. A global score was calculated ranging from 0 (the lowest level of optimism) to 24 (the highest level of optimism). No missing data were allowed for the calculation of this score.

### Other data

Other recorded data included socio-demographic variables, i.e. age, gender, professional activity, family situation, level of education, place of residence, use of psychotropic drugs; and clinical data, i.e. interventions performed during hospital stay, primary chronic disease diagnosis category motivating the hospital stay, and participating center.

### Statistical analysis

#### Imputation of missing data

We calculated the percentage of items not answered to impute the missing data for each dimension of the SF36 and for the STAI score. The percentage of missing data was low, between 0.26% and 5%. The missing data were considered to be missing completely at random (MCAR), and thus, missing data were imputed with the simple mean of responded items per dimension [40,41].

### Descriptive analysis

First, we selected factors to be adjusted for in the assessment of the relation between HRQOL scores and patient characteristics (socio-demographic and medical data) using a Multiple ANalysis Of VAriance (MANOVA). Variables with a significant relation at an alpha risk of 10% were retained for multivariate analysis. Associations between HRQOL and both anxiety and optimism were evaluated using linear regression models.

Then, an ANalysis Of VAriance (ANOVA) was performed to examine the relation between HRQOL and the personality traits for each SF36 dimension score. Scores of optimism and anxiety were categorized in 4 classes using median and quartiles. To explain the contribution of these two factors to HRQOL, 3 models were built: firstly, a model assessing the effect of anxiety on HRQOL adjusted for confounders; secondly, a model evaluating the effect of optimism on HRQOL adjusted for confounders; and thirdly, a model assessing the effect of both anxiety and optimism on HRQOL, and their interaction, adjusted for confounders.

All analyses were performed using SAS version 9.3 (SAS Institute Inc., Cary, NC, USA).

### Sample size and power of statistical analysis

Among the 1657 patients included in the SATISQOL cohort, 1529 completed the SF36 questionnaire at 6 months (response rate = 92%). This sample size would make it possible to detect a difference of 5 points in HRQOL at an alpha risk of 0.006 (0.05/8, using Bonferroni correction for multiple tests) and a power of 95% (at a standard deviation of 20 points) to 85% (at a standard deviation of 25 points).

## Results

### Study population

Of the 1529 patients included in the study, most were aged over 55 years old (61.2%) and were no longer professionally active (67.5%). The majority lived at home (98.2%) and did not live alone (81.2%). The intervention performed during the index hospitalization was often surgery (50.5%). Only 20.4% had taken psychotropic treatments. Descriptive data are presented in Table 1.

**Table 1:** Characteristics of the study population

**Variables**	**Category**	**n (%)**
**Center**		
	1	314 (20.5)
	2	617 (40.4)
	3	598 (39.1)
**Gender**		
	Men	886 (58.1)
	Women	639 (41.9)
**Age**		
	<45	328 (21.5)
	45-55	264 (17.3)
	55-65	461 (30.1)
	>65	476 (31.1)
**Professional activity**		
	Yes	459 (32.5)
	No	953 (67.5)
**Family situation**		
	Alone	270 (18.8)
	Not alone	1164 (81.2)
**Study level**		
	Primary school	335 (24.3)
	Middle and high school	751 (54.7)
	University	288 (21.0)
**Place of residence**		
	At home	1405 (98.2)
	Institution	14 (1.0)
	Other	11 (0.8)
**Intervention**		
	Surgery	755 (50.5)
	Interventional	299 (20.0)
	Medical	440 (29.5)
**Diagnosis**		
	ENT Ophtalmology	126 (8.4)
	Cardiovascular	33 (2.2)
	Gastroenterology	370 (24.6)
	Endocrinology	125 (8.3)
	Neurology	267 (17.7)
	Oncology	219 (14.6)
	Pneumology	129 (8.6)
	Rheumatology	39 (2.6)
	Urology Nephrology	174 (11.6)
	Others	24 (1.4)
**Use of psychotropic drugs**		
	Yes	291 (20.4)
	No 1135	(79.6)

Table 2 shows the distribution of diagnosis category, interventions, and optimism and anxiety scores in each center. In center 3, the category of diagnosis was mainly rheumatology (24.1%) or ear/nose/throat (ENT) and ophthalmology (21.1%), while in centers 1 and 2, the main diagnosis was cardiovascular (20.1% and 38.7% respectively), endocrinology (25.9% and 16.4% respectively) or respiratory disease (24.3% in center 1). The interventions performed during hospitalisation were: surgery for 78.5% of patients in center 3, and for only about 30% of patients in the other centers. In center 3, patients were more optimistic, with a score >17 observed in 32.3%. They were also less anxious, with a score >41 for only 19.4%.

**Table 2:** Distribution of diagnosis, intervention, anxiety and optimism scores in each center

**Variables**	**Category**	**Center 1**	**Center 2**	**Center 3**
		**n (%)**	**n (%)**	**n (%)**
**Diagnosis**				
	ENT			
	Ophthalomology	0 (0.0)	0 (0.0)	126 (21.1)
	Cardiovascular	62 (20.1)	232 (38.7)	76 (12.7)
	Gastroenterology	28 (9.1)	178 (11.8)	61 (10.2)
	Endocrinology	80 (25.9)	98 (16.4)	41 (6.9)
	Neurology	0 (0.0)	2 (0,3)	37 (6.2)
	Oncology	0 (0.0)	1 (0,2)	32 (5.4)
	Pneumology	75 (24.3)	38 (6,3)	12 (2.0)
	Rheumatology	26 (8.4)	3 (0,5)	144 (24.1)
	Urology Nephrology	38 (12.3)	45 (7.5)	46 (7.7)
	Others	0 (0.0)	2 (0.3)	22 (3.7)
**Intervention**				
	Surgery *	104 (33.7)	182 (30.9)	469 (78.5)
	Interventional **	32 (10.4)	202 (34.4)	65 (10.9)
	Medical ***	173 (56.0)	204 (34.7)	63 (10.5)
**Anxiety score**				
**(STAI)**	4-17	75 (23.9)	157 (25.4)	176 (29.4)
	17-29	84 (26.7)	129 (20.9)	140 (23.4)
	29-41	68 (21.7)	154 (25.0)	166 (27.8)
	41-80	87 (27.7)	177 (28.7)	116 (19.4)
**Optimism score**				
**(LOTR)**	0-13	98 (31.2)	175 (28.4)	135 (22.6)
	13-15	49 (15.6)	117 (19.0)	109 (18.2)
	15-17	80 (25.5)	180 (29.1)	161 (26.9)
	17-24	87 (27.7)	145 (23.5)	193 (32.3)

* Surgery : patients hospitalized for surgery in connection with the chronic disease.

** Interventional : patients hospitalized for non-surgical intervention (e.g. interventional radiology or non invasive ventilation) in connection with the chronic disease.

*** Medical : patients hospitalized for drug therapy in connection with the chronic disease.

### Scores of HRQOL, anxiety and optimism

The mean HRQOL score was between 48.7 and 66.6 for the 8 dimensions (Table 3). The mean anxiety score was 30.1, and the mean optimism score was 15.0.

**Table 3:** Description of dimensions of quality of life and psychological measures

	**n**	**Mean**	**Standard deviation**	**Median**
**SF36 score dimensions (0-100)**				
**Physical Functioning score**	1486	66.6	28.6	75.0
**Role Physical score**	1491	48.7	43.2	50.0
**Bodily Pain score**	1511	57.0	27.5	52.0
**Vitality score**	1479	47.0	21.6	50.0
**Social Functioning score**	1525	65.0	27.2	62,5
**Role Emotional score**	1463	53.8	44.8	66,7
**Mental Health score**	1470	59.7	21.6	60.0
**General Health score**	1429	50.6	23.4	52.0
**Anxiety score (4-80)**	1454	30.1	16.2	29.0
**Optimism score (0-24)**	1398	15.0	2.9	15.0

### Relation between HRQOL and personality traits

**Figure 2:**
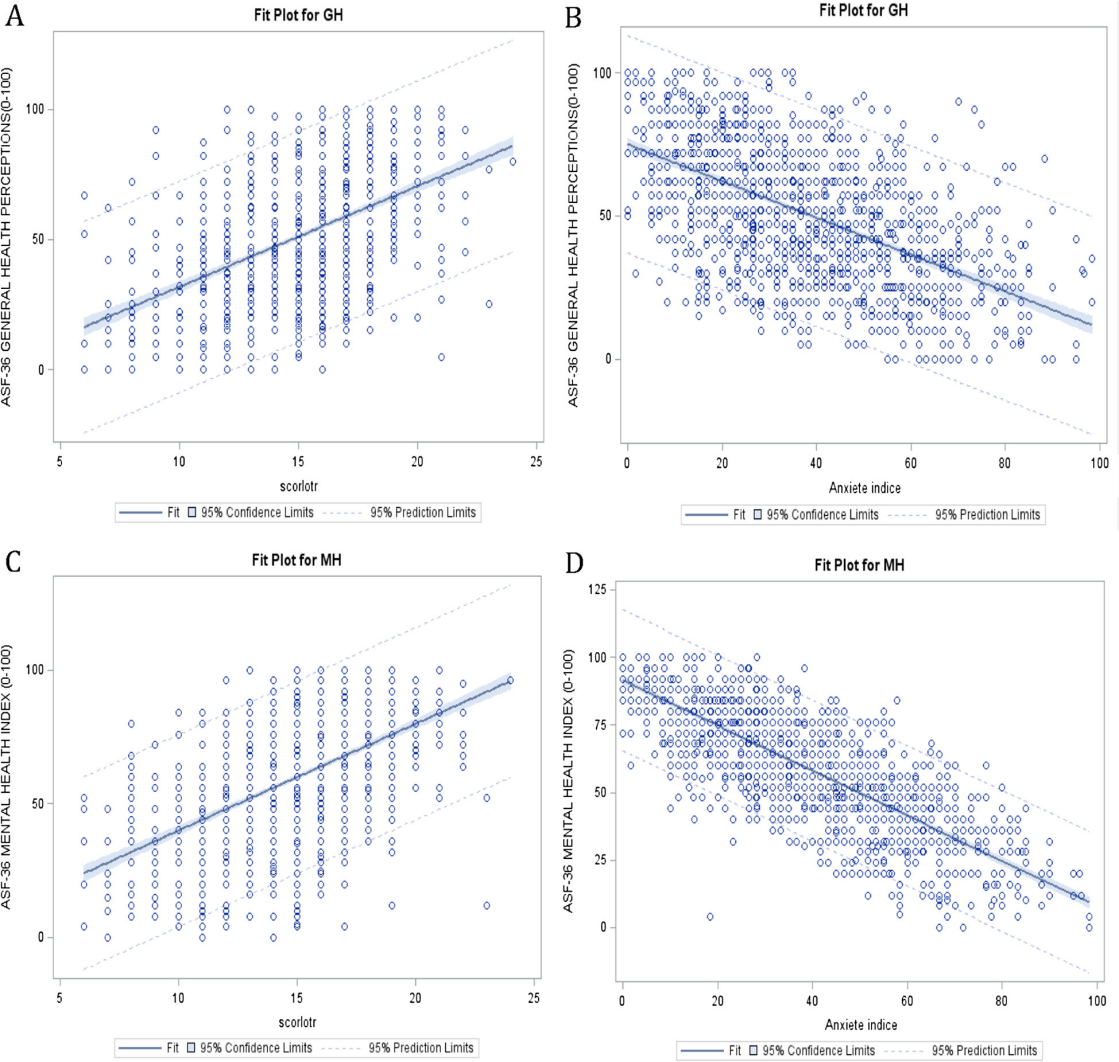
**Relation between anxiety or optimism and MH and GH dimensions scores of SF36. A**. Relation between optimism and GH dimension score of SF36 (β : 3.86; R ^2^ : 0.228). **B**. Relation between anxiety and GH dimension score of SF36 (β: -1.39; R^2^ : 0.621). **C**. Relation between optimism and MH dimension score of SF36 (β: - 3.99; R ^2^ : 0.287). **D**. Relation between anxiety and MH dimension score of SF36 (β: - 1.07; R ^2^ : 0.311).

There was a significant relation (p < 0.0001) between each dimension of SF36 and the optimism and anxiety scores. HRQOL decreased with increasing levels of anxiety and increased with increasing level of optimism, including for dimensions MH and GH (Figure 2).

The variables of adjustment retained for the multivariate models significantly related to HRQOL were: center, age, gender, intervention, level of education, and use of psychotropic drugs. Diagnosis had 10 categories and we did not use this variable in the multivariate model. Likewise, professional activity was also excluded from the multivariate model, as it was significantly related to age and level of education. We did not retain family situation because most of the patients were not living alone.

### Model 1 (HRQOL and anxiety)

The HRQOL score increased for all dimensions of SF36 when the level of anxiety decreased (p < 0.0001) in this model (Tables 4 and 5). The difference in mean HRQOL scores between patients with the highest and lowest levels of anxiety was between 16 and 43.5.

**Table 4:** Association of anxiety or optimism with quality of life (dimensions PF, RP, BP and VT of SF36)

		**Physical functioning score**			**Role physical score**			**Bodily pain score**			**Vitality score**		
		**M1**	**M2**	**M3**	**M1**	**M2**	**M3**	**M1**	**M2**	**M3**	**M1**	**M2**	**M3**
**Anxiety score**													
	4-17*	16.0		17.3	32.4		35.3	22.9		15.8	28.6		28.3
	17-29*	15.1		16.1	28.5		29.4	19.9		11.4	22.1		21.3
	29-41*	6.8		7.8	16.7		17.7	8.9		- 2.7	11.8		10.8
	41-80**	*(54.2)*		*(55.2)*	*(26.2)*		*(26.9)*	*(42.2)*		*(45.8)*	*(28.1)*		*(29.8)*
	p-value	*<0,0001*		*<0,0001*	*<0,0001*		*<0,0001*	*<0,0001*		*<0,0001*	*<0,0001*		*<0,0001*
**Optimism score**													
	0-13*		-14.6	- 3.1		- 24.9	- 3.8		- 16.8	- 13.0		- 19.5	- 5.8
	13-15*		- 6.9	2.2		- 18.5	- 4.5		- 9.9	- 9.1		- 14.3	- 0.5
	15-17*		- 4.1	0.6		-12.7	- 2.5		- 4.4	-2.6		- 7.3	- 0.1
	17-20**		*(69.2)*	*(65.6)*		*(57.3)*	*(50.2)*		*(61.4)*	*(58.1)*		*(51.9)*	*(46.5)*
	p-value		*<0,0001*	*0.0038*		*<0,0001*	0.0522		*<0,0001*	0.0012		*<0,0001*	*0,0001*
**Interaction Anxiety score and Optimism score**	p-value			*0.9872*			0.1792			0.2807			*0.3709*

*Figure in each cells indicates he mean difference with reference class value**.

**reference class : (absolute score value).

M1 (Model 1) : Relation between anxiety and quality of life.

M2 (Model 2) : Relation between optimism and quality of life.

M3 (Model 3) : Relation between anxiety, optimism and quality of life.

All models were adjusted for center, age, gender, level of education, use of psychotropic drugs, p significant <0.006.

Dimensions of SF36 score : PF (Physical Functionning), RP (Role Physical), BP (Bodily Pain), VT (Vitality).

Anxiety score: STAI score <33 corresponds to the lowest level of anxiety and >51 to the highest level of anxiety.

Optimism score : LOT-R score <13 corresponds to the lowest level of optimism and >17 to the highest level of optimism.

**Table 5:** Association of anxiety or optimism with quality of life (dimension SF, RE, MH and GH of SF36)

		**Social functioning score**			**Role emotional score**			**Mental health score**			**General health score**		
		**M1**	**M2**	**M3**	**M1**	**M2**	**M3**	**M1**	**M2**	**M3**	**M1**	**M2**	**M3**
**Anxiety score**													
	4-33*	34.4		29.2	43.5		44.5	37.4		37.2	26.9		23.8
	17-29*	30.1		22.1	38.9		36.3	31.0		28.7	20.4		17.1
	29-41*	17.4		10.7	23.4		25.3	17.6		15.3	10.6		6.2
	41-80**	*(40.8)*		*(44.4)*	*(23.7)*		*(25.2)*	*(36.9)*		*(38.5)*	*(32.1)*		*(35.2)*
	p-value	*<0,0001*		*<0,0001*	*<0,0001*		*<0,0001*	*<0,0001*		*<0,0001*	*<0,0001*		*<0,0001*
**Optimism score**													
	0-13*		-23.0	- 12.7		- 29.8	- 6.7		- 24.6	- 6.2		- 23.4	- 11.8
	13-15*		- 15.0	- 4.7		- 26.4	- 6.1		- 16.2	- 1.1		- 16.4	- 4.7
	15-17*		- 6.7	- 2.6		- 13.5	- 4.2		- 9.1	- 1.9		- 9.9	- 4.0
	17-20**		*(70.3)*	*(64.9)*		*(64.2)*	*(55.9)*		*(68.2)*	*(61.1)*		*(57.1)*	*(52.1)*
	p-value		*<0,0001*	*<0,0001*		*<0,0001*	0.0069		*<0,0001*	*<0,0001*		*<0,0001*	*<0,0001*
**Interaction Anxiety score and Optimism score**	p-value			*0.0021*			0.3142			0.0365			*0.0300*

*Figure in each cells indicates he mean difference with reference class value **.

**reference class : (absolute score value).

M1 (Model 1) : Relation between anxiety and quality of life.

M2 (Model 2) : Relation between optimism and quality of life.

M3 (Model 3) : Relation between anxiety, optimism and quality of life.

All models were adjusted for center, age, gender, level of education, use of psychotropic drugs, p significant <0.006.

Dimensions of SF36 score : SF, (Social Functionning) RE (Role Emotional), MH (Mental Health), GH (General Health).

Anxiety score: STAI score <33 corresponds to the lowest level of anxiety and >51 to the highest level of anxiety.

Optimism score : LOT-R score <13 corresponds to the lowest level of optimism and >17 to the highest level of optimism.

### Model 2 (HRQOL and optimism)

The HRQOL score increased for all dimensions of SF36 with increasing level of optimism (< 0.0001) (Tables 4 and 5). The difference in mean HRQOL score between patients with the lowest and the highest level of optimism was between 14.6 and 29.8.

### Model 3 (HRQOL and anxiety and optimism)

The HRQOL score for all dimensions of the SF36 increased when the level of anxiety decreased (p < 0.0001) (Tables 4 and 5). The difference in mean HRQOL score between the patients with the lowest and the highest level of anxiety was between 15.8 and 44.5.

The HRQOL score increased with increasing level of optimism (p < 0.006) in the model for all dimensions of SF36 except RP (Tables 4 and 5). The difference in mean HRQOL score between the patients with the lowest and the highest level of optimism was between 3.1 and 12.7.

**Figure 3:**
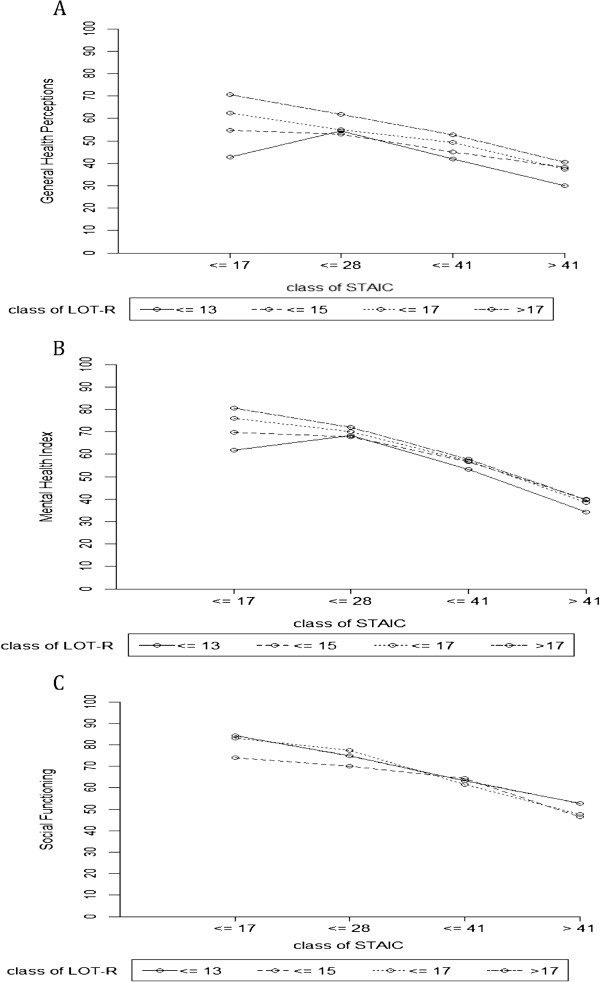
**Interaction between optimism and anxiety for the SF, MH, GH dimensions scores of SF36. A**. for the GH dimension score of SF36 (p = 0.0049; R ^2^ = 0.30). **B**. for the MH dimension score of SF36 (p = 0.0008; R ^2^ = 0.53). **C**. for the SF dimension score of SF36 (p = 0.0001; R ^2^ = 0.31).

In this model, interaction between anxiety and optimism was significant for the SF dimension (p = 0.0021). For the dimensions MH (p = 0.0365) and GH (p = 0.0300), interaction was not significant at the significance level with Bonferroni correction (alpha risk of 0.006). Then, a model for each significant dimension was constructed to study the interaction between optimism and anxiety, which remained significant for the dimensions GH (p = 0.0049; R ^2^ 0.30), MH (p = 0.0008; R ^2^ = 0.53) and SF (p = 0.0001; R ^2^ = 0.31) (Figure 3). For patients with a low level of anxiety, the level of optimism strongly influenced the level of HRQOL for this dimension. A correlation existed between anxiety and optimism (R ^2^ = 0.39).

We also investigated the composite scores. The Physical Composite Score (PCS) increased when the level of anxiety decreased (from 41.7 at the highest level of anxiety to 64.2 at the lowest level of anxiety) (p < 0.0001). Similarly, the PCS increased with the level of optimism (from 63.4 at the lowest level to 71.2 at the highest level of optimism) (p = 0.0024). The interaction between anxiety and optimism was not significant (p = 0.4705). The Mental Composite Score (MCS) increased when the level of anxiety decreased (from 39.7 at the highest level of anxiety to 67.8 at the lowest level of anxiety) (p < 0.0001), and increased with the level of optimism (from 52.8 at the lowest level to 59.4 at the highest level of optimism) (p < 0.0001). The interaction between anxiety and optimism was not significant, at the significance level with Bonferroni correction (p = 0.0476).

For this model, as for the other models, HRQOL score was significantly higher for all dimensions except MH in center 3 than in the other centers (p < 0.03), and was significantly better in men for the dimensions PF (p = 0.013), BP (p = 0.014), RE (p = 0.02) and MH (p = 0.006). HRQOL score was significantly higher for younger vs older patients for every dimension except BP and MH (p < 0.05). When the intervention performed during hospitalization was surgery, HRQOL was better, except for BP dimension (p < 0.05). HRQOL was better among patients with a high level of education for the dimensions PF (p = 0.0011), RP (p = 0.0009), BP (p = 0.0013) and RE (p = 0.0014). Patients who were taking psychotropic drugs had a lower level of HRQOL for each dimension (p < 0.01).

## Discussion

### Main results of our study

To the best of our knowledge, this is the first study to specifically examine the relationship between HRQOL and optimism and trait anxiety. By modeling both factors separately and simultaneously, we showed significant associations between personality traits and the HRQOL of patients with chronic disease, especially the effect of anxiety on HRQOL scores, whatever the dimension. These results suggest that for patients hospitalized for chronic disease, a high level of anxiety is significantly associated with lower HRQOL for all dimensions. On the other hand, HRQOL increased with increasingly level of optimism, although the relation was less marked than for anxiety. Therefore, anxiety could be a better determinant of HRQOL in this population.

Furthermore, we took into account the interaction between optimism and anxiety, which was not the case in other published studies. In our study, we found a moderator-interaction effect (optimism) that affects the strength of the relation between a predictor variable (anxiety-trait) and an outcome variable (HRQOL) for some dimensions, in particular for the social dimension, which has never studied before. Thus, at low levels of anxiety, the effect of optimism on HRQOL was amplified. Optimism reduces the negative impact of anxiety on HRQOL.

### Data in the literature

Our results are in agreement with those of previously published studies. First of all, the correlation between the scores of anxiety and optimism was lower in our population than in the validation of the LOTR (R ^2^ = 0.53) [13], but higher than in other studies [23,35].

When looking at the HRQOL scores in the general population [39], the scores in our population were lower for anxious patients (between 25 and 55) and more than 50 for less anxious patients (the main score in general population was comprised between 68 and 82 according to the dimension). Similarly, for patients who had a high level of optimism, the HRQOL scores were lower than in the general population, ranging from 50 to 70.

Furthermore, most studies have focused on the relation between optimism and HRQOL, and have recommended the use of the questionnaire on optimism in the evaluation of HRQOL or to propose adapted care [4,19,20]. In this view, pessimism could decrease HRQOL [21,23]. However, although the effect of optimism has often been raised in the evaluation of HRQOL, its impact remains controversial. For women with breast cancer followed-up for 2 years, optimism was not able to predict HRQOL 2 years after the primary operation [26]. Another study reported that after a recent diagnosis of cancer, optimism was correlated with anxiety and HRQOL, but was not a significant predictor at initial diagnosis when other variables like age, functional status, spiritual well-being, depression and anxiety were entered in the model [42].

Finally, fewer studies exist examining the relation between trait anxiety and HRQOL. Most have explored state anxiety, and not trait anxiety as in our study. These studies underlined the effect of anxiety and depression on HRQOL [3,29,32,34], although some reports revealed that interventions like surgery could increase the level of anxiety and depression without decreasing HRQOL. We did not take state anxiety and depression into account in our study. However, we adjusted our analyses for the use of psychotropic drugs, and noted that patients taking psychotropic therapy had a lower HRQOL.

### Study limitations

Our study suffers from several limitations. Previous studies have suggested that HRQOL may be different according to the type of chronic disease [43,44]. It can also be estimated differently depending on whether a specific or generic questionnaire is used. For example, in rheumatology, anxiety was more correlated with a specific questionnaire than with the SF36 [45]. Furthermore, we could not take comorbidity into account, and the detriment to HRQOL could be greater in patients with two or more concomitant chronic diseases [6]. Lastly, we were not able to introduce diagnosis into the model, because of the multiplicity of categories. However, we adjusted for the intervention performed during hospitalization, which at least partially reflects the diagnosis.

### Impact and strengths of our study

The strongpoint of our study was to simultaneously study the role of anxiety and optimism in the evaluation of HRQOL.

We had a large multicenter cohort of patients, with a large panel of chronic diseases and thus with high statistical power. A further strongpoint of our study was to evaluate quality of life for many types of disease, using the same generic questionnaire, because most HRQOL studies have investigated a specific disease. Although HRQOL was different between centers participating in the study, we adjusted for this variable, thereby minimizing the potential for bias. Thanks to our use of validated generic questionnaires, our results can be generalized to a large population of patients for many types of chronic disease.

Furthermore, the interaction between trait anxiety and optimism has never yet been studied, even though most studies have reported an effect of optimism on HRQOL. We found that the effect of anxiety on HRQOL was more marked than the effect of optimism. In order to interpret this result appropriately, we must consider trait anxiety as an emotional component, associated with psychological symptoms, whereas optimism is mainly composed by cognitions associated with a perception of the world. Thus, the greater impact of anxiety on quality of life could be due in part to the difference between evaluating an emotional component and a cognitive component.

Although it does not appear essential to measure optimism systematically, it nonetheless seems important to estimate anxiety in the evaluation of HRQOL, because its effect is major. Therefore, we recommend the use in research practice of the trait anxiety questionnaire in future studies evaluating HRQOL, which could be completed by a measure of coping strategies for chronic diseases. The evaluation of coping strategies such as positive reinterpretation, humor associated with optimism or rumination, dramatization associated with more anxiety, would be a useful complement to future studies of the impact of personality traits on HRQOL.

## Conclusion

The relation between psychological factors and HRQOL has often been evoked in patients with chronic disease. This is the first study of the relation between HRQOL and the character traits like optimism and anxiety, as well as their interaction, performed in a large cohort of patients with many types of chronic diseases. Optimism and trait anxiety appeared to be significantly correlated with HRQOL. Furthermore, an interaction existed between the trait anxiety and optimism for some dimensions of SF36. Accordingly, HRQOL for less anxious patients was heavily influenced by the level of optimism. Although some studies have revealed the impact of optimism on HRQOL, trait anxiety seems to be a better determinant of HRQOL.

These results could have implications for future studies of HRQOL in patients with chronic diseases. It will be necessary to take account of these results and to evaluate the psychological factors when interpreting HRQOL in large populations of patients followed up for chronic disease.

## Abbreviations

HRQOL: Health Related Quality Of Life; SF36: Short form 36; PF: Physical functioning; RP: Role physical; BP: Bodily pain; VT: Vitality; SF: Social functioning; RE: Role emotional; MH: Mental health; GH: General health; STAI: State trait anxiety inventory; LOT-R: Life orientation test revised.

## Competing interests

The authors have no potential conflict of interest.

## Authors’ contributions

SK participated in the conception and design of the study, acquisition of data, analysis and interpretation of data, drafting of the manuscript and final approval of the manuscript to be published. CB participated in the conception and design of the study, acquisition of data, analysis and interpretation of data, drafting of the manuscript and final approval of the manuscript to be published. AA participated in the analysis and interpretation of data and had given final approval of the manuscript to be published. GB participated in the acquisition of data and final approval of the manuscript to be published. ES participated in the conception and design of the study, revising the manuscript critically for important intellectual content and final approval of the manuscript to be published. PA participated in the conception and design of the study, acquisition of data, revising the manuscript critically for important intellectual content and final approval of the manuscript to be published. FG participated in the conception and design of the study, acquisition of data, revising the manuscript critically for important intellectual content and final approval of the manuscript to be published. MM participated in the conception and design of the study, acquisition of data, analysis and interpretation of data, drafting of the manuscript and final approval of the manuscript to be published. All authors read and approved the final manuscript.
